# Activated spinal astrocytes contribute to the later phase of carrageenan-induced prostatitis pain

**DOI:** 10.1186/s12974-019-1584-3

**Published:** 2019-10-25

**Authors:** Guo-Chuang Deng, Ming Lu, Ya-Yu Zhao, Ying Yuan, Gang Chen

**Affiliations:** 1 Key Laboratory of Neuroregeneration of Jiangsu and Ministry of Education, Co-innovation Center of Neuroregeneration, Nantong University, Nantong, China; 2 Department of Urology, The Second Affiliated Hospital of Nantong University (The First People’s Hospital of Nantong), Nantong, China; 3 Department of Tissue and Embryology, Medical School of Nantong University, Co-innovation Center of Neuroregeneration, Nantong University, Nantong, China; 4 Department of Anesthesiology, Affiliated Hospital of Nantong University, Nantong, China

**Keywords:** Prostatodynia, Microglia, Astrocyte, Connexin43, CXCL1

## Abstract

**Background:**

Prostatodynia is the main symptom of chronic prostatitis and the main reason that patients go to the hospital for treatment. Although a variety of factors, including inflammatory immune response, nervous system sensitization, and psychological factors, have been shown to play important roles in the induction and development of chronic pain in prostatitis, the underlying cause of chronic prostatodynia maintenance in prostatitis patients remains unclear.

**Methods:**

A mouse model of chronic prostatitis induced by carrageenan injection was used. The von Frey test was used to measure pain behavior. The microglial and astrocyte activations were immunohistochemically demonstrated with antibodies against Iba1 and GFAP. The expression of cytokine or signaling pathway was detected by enzyme-linked immunosorbent assay (ELISA) and Western blotting.

**Results:**

In this study, we provide several lines of evidence to demonstrate that activated spinal astrocytes contribute to the later phase (5 weeks after carrageenan injection) of carrageenan-induced prostatitis pain. First, activation of spinal astrocytes but not microglia was found in the spinal cord dorsal horn at 5 weeks. Second, intrathecal injection of the astroglial toxin L-2-Aminoadipate acid (L-AA) but not microglial inhibitor minocycline reduced mechanical allodynia at 5 weeks. Third, chronic prostatitis induced a profound and persistent upregulation of connexin-43 hemichannels in spinal astrocytes, and spinal injection of the connexin-43 inhibitor carbenoxolone (CBX) effectively reduced pain symptoms. Fourth, increased expression and release of chemokine C-X-C motif ligand 1 (CXCL1) in the spinal dorsal horn and intrathecal injection of a CXCL1 neutralizing antibody or the CXCR2 (a major receptor of CXCL1) antagonist SB225002 significantly reduced mechanical allodynia at 5 weeks.

**Conclusions:**

In this study, we found that a novel mechanism of activated spinal astrocytes plays a crucial role in maintaining chronic prostatitis-induced persistent pain via connexin-43-regulated CXCL1 production and secretion.

## Background

Chronic prostatitis/chronic pelvic pain syndrome (CP/CPPS), also known as NIH category III prostatitis [1], is one of the most common urinary system diseases in young and middle-aged men. An estimated 9–16% of men experience the symptoms of prostatitis in their lifetime [2, 3]. Clinical studies have found that prostatodynia is the main symptom of CP/CPPS and the main reason that patients go to the hospital for treatment [4]. Prostatodynia is a visceral pain with pelvic floor muscle dysfunction. The pain is usually not limited to the prostate but is present in most areas of the sacral nerve [5]. In addition, some patients feel pain even after the disappearance of prostatitis. Although a variety of factors, including inflammatory immune response, nervous system sensitization, and psychological factors, have been shown to play important roles in the induction and development of chronic pain in prostatitis, the underlying cause of chronic prostatodynia maintenance in prostatitis patients remains unclear [6]. Furthermore, in clinical practice, although various analgesic drugs have been tested, prostatodynia is often not resolved during the treatment of CP/CPPS [7]. Therefore, there is an urgent need for more effective treatment methods for patients with prostatodynia.

Current research confirms that tissue and nerve injury-induced synaptic plasticity in the peripheral and central nervous systems is the main cause of chronic pain and extensive pain outside the initial injury site [8]. Previous studies have also shown that the persistence of prostate pain may be related to the conduction pathway and abnormal neuromodulation in the L6~S2 spinal segment, which dominates the prostate [9]. After stimulating the bladder and prostate with acetic acid or formalin, the number of c-fos-positive cells in the lumbosacral spinal cord was significantly increased [10], indicating that the lumbosacral spinal cord is involved in the neural circuit of prostate pain. Furthermore, immunohistochemistry and pharmacological blockade experiments have also illustrated that CP/CPPS can activate microglia in the rat L5-S2 spinal cord through the P2X7 receptor [11, 12] and that inhibition of microglia activation can alleviate persistent prostate pain [11]. There is increasing evidence that spinal microglia and astrocytes play important roles in chronic pain sensitization by releasing cellular mediators such as cytokines, chemokines, and growth factors [13–15]. Spinal astrocytes exhibit immunological and endocrine pathological changes in neuropathy, produce paracrine and endocrine proinflammatory and chemotactic factors via multiple mechanisms, including ERK phosphorylation and connexin 43 upregulated, and are closely related to the conduction, maintenance, and generalization of pain [16–18]. However, whether astrocyte proliferation and activation are present in chronic prostate pain remains unclear. In addition, previous studies of prostate pain maintenance have focused on early phases that occur in the first 1–4 weeks after injury [9, 11, 19], and the specific role of glial cells in maintaining late-phase (> 5 weeks) prostate pain remains unclear.

Carrageenan is a polysaccharide which is commonly used to induce inflammation pain [20, 21]. The carrageenan-induced prostatitis model could mimic the NIH category IIIA prostatitis, where inflammation, and not bacterial infection, is believed to be the major cause of CP/CPPS [19]. In this study, we have mainly focused on late stage of prostate pain (5 weeks after carrageenan-induced CP/CPPS) and hypothesized that carrageenan-induced CP/CPPS in mice would lead to astrocyte activation at the level of the spinal cord and that this activation is critical for the maintenance of prostate pain.

## Methods

### Reagents

We obtained carrageenan (catalog: C1013, Predominantly κ and lesser amounts of λ carrageenan), minocycline (catalog: M9511), U0126 (catalog: U120), L-2-Aminoadipate acid (L-AA; catalog: A7275), and carbenoxolone (CBX; catalog: C4790) from Sigma-Aldrich, SB 225002 (catalog: 182498-32-4) from Tocris, and CXCL1 neutralizing antibody (catalog: A00533) from Boster.

### Animals and surgery

**Fig. 1:**
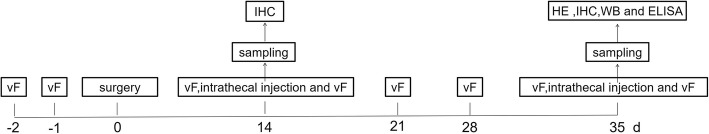
Experimental time flow. Pain threshold measurement, using a series of von Frey hairs with logarithmically increasing stiffness (0.02–2.56 g, Stoelting), was performed day − 2 and day − 1 prior to, as well as on day 14, day 21, day 28, and day 35 upon surgery (intraprostatic injection of 1% carrageenan (20 μl), day 0). The effects of intrathecal injection of different reagents on pain threshold were measured on day 14 or 35 after surgery. Some of sham and CP/CPPS mice were sacrificed for spinal cord slices for immunostaining on day 14. Others were sacrificed for prostate sampling for histology examination (H&E), spinal cord slices for immunostaining, spinal cord dorsal horn structures isolation for CXCL1 measurement by ELISA, and for pERK and ERK expression measurement by Western blot on day 35

All animal procedures were in full compliance with the Directive of the National Institutes of Health and approved by the Institutional Animal Care & Use Committee (IACUC) of Nantong University. Adult CD-1 mice (male, 25–30 g) were purchased from the Experimental Animal Center of Nantong University. The numbers of mice used in different experiments were shown in Additional file 1: Table S1. The schematic illustration of the experimental design is shown in Fig. 1. In brief, to produce model of chronic prostatitis, mice were anesthetized using 5% isoflurane and maintained at 2–3% v/v isoflurane. After shaving and disinfecting the lower abdomen above the penis, an incision (2 cm) was made on the skin and abdominal wall to expose the ventral lobe of the prostate [19]. A sterile suspension of 1% carrageenan (20 μl) was injected into both right and left ventral lobes of the prostate gland using a 30G needle (Additional file 1: Figure S1). For the sham group, carrageenan solution was replaced by an equal volume of saline. The wound on the muscle layer and skin were closed using sterilized absorbable suture material. Body weight and food and water consumption by animals in each group were measured once each week. No death or abnormal behavior was observed during the course of the study. For intrathecal injection, spinal cord puncture was made with a 30G needle between the L5 and L6 level to deliver reagents (10 μl) to the cerebral spinal fluid.

### Behavioral testing

All the behaviors were tested blindly as we described previously [22]. Mice were habituated to the environment for at least 2 days before the testing. For testing mechanical sensitivity, we confined mice in boxes placed on an elevated metal mesh floor and stimulated the lower abdominal area (near the suprapubic) with a series of von Frey hairs with logarithmically increasing stiffness (0.02–2.56 g, Stoelting). We determined the 50% sharp retraction of the abdomen by Dixon’s up-down method.

### Histological and immunohistochemistry analysis

As we reported previously [23], mice were deeply anesthetized with isoflurane and perfused through the ascending aorta with saline, followed by 4% paraformaldehyde. To examine the prostate inflammation histologically, the ventral lobes of the prostate were subjected to routine staining with hematoxylin and eosin and evaluated with a light microscope. Quantitation of the degree of inflammation in prostate was according to previous literature [24]. For immunohistochemistry, the L6–S1 spinal cord segments were cut in a cryostat (30 μm, free-floating) and processed for immunohistochemistry. The sections were first blocked with 2% goat serum for 1 h at room temperature and incubated overnight at 4 °C with the following primary antibodies: Iba-1 antibody (1:500, rabbit; Wako, catalog: 019-19741), GFAP antibody (1:500, mouse; Millipore Bioscience Research Reagents, catalog: MAB360), and Cx43 antibody (1:500, rabbit; Sigma, catalog: C6219), The sections were then incubated for 2 h at room temperature with Alexa 488-anti-rabbit (1:1000, Jackson ImmunoResearch, catalog:111-545-003). For double immunofluorescence, sections were incubated with a mixture of monoclonal and polyclonal primary antibodies, followed by a mixture of cyanine 3-anti mouse (1:1000, Jackson ImmunoResearch, catalog: 115-165-003) or Alexa 488-anti-rabbit (1:1000, Jackson ImmunoResearch, catalog: 111-545-003). The stained sections were examined with a Nikon fluorescence microscope, and images were captured with a CCD Spot camera. We collected eight spinal cord sections from each mouse for quantification of immunofluorescence. The intensity of fluorescence in spinal cord superficial dorsal horn (laminae I–III) was measured by a blinded observer using NIH ImageJ software from a rectangular region (schematic diagram is shown in Additional file 1: Figure S2) and normalization with background subtraction. The region remained consistent between sections.

### Western blot

Protein samples were prepared from spinal cord dorsal horn tissues, and 20 μg of proteins was separated on SDS-PAGE gel (4–15%; Bio-Rad) as previously reported [13]. After the transfer, the blots were incubated overnight at 4 °C with following primary antibodies: phospho-ERK (pERK) antibody (1:1000, rabbit, Cell signaling, catalog: 9101), total ERK (tERK) antibody (1:1000, rabbit; Cell signaling, catalog: 9102), and GAPDH antibody (1:2000, mouse, Proteintech, catalog: 60004-1-lg). These blots were further incubated with HRP-conjugated secondary antibody, developed in ECL solution (Pierce), and the chemiluminescence was revealed by Bio-Rad ChemiDoc XRS for 1–5 min. Specific bands were evaluated by apparent molecular sizes. The intensity of the selected bands was analyzed using NIH ImageJ software. Western gel images have been cropped for presentation. Full size gel images are presented in Additional file 1: Figure S4.

### Enzyme-linked immunosorbent assay

Mouse CXCL1 ELISA kits were purchased from R&D Systems (catalog: MKC00B). As we described previously [16], spinal cord dorsal horn tissues were homogenized in a lysis buffer containing protease and phosphatase inhibitors and cerebrospinal fluid was collected from cisterna magna 3 h after intrathecal injection of CBX or PBS. For each ELISA assay in a 96-well plate, 50 μg of proteins or 5 μl of CSF was used. ELISA was conducted according to the manufacturer’s instructions. The standard curve was included in each experiment.

### Statistical analyses

All data were expressed as mean ± SEM and analyzed using Student’s *t* test or two-way ANOVA, followed by post hoc Bonferroni test. The criterion for statistical significance was *P* < 0.05.

## Results

### Inflammation and hyperalgesia caused by prostate injection of carrageenan

**Fig. 2:**
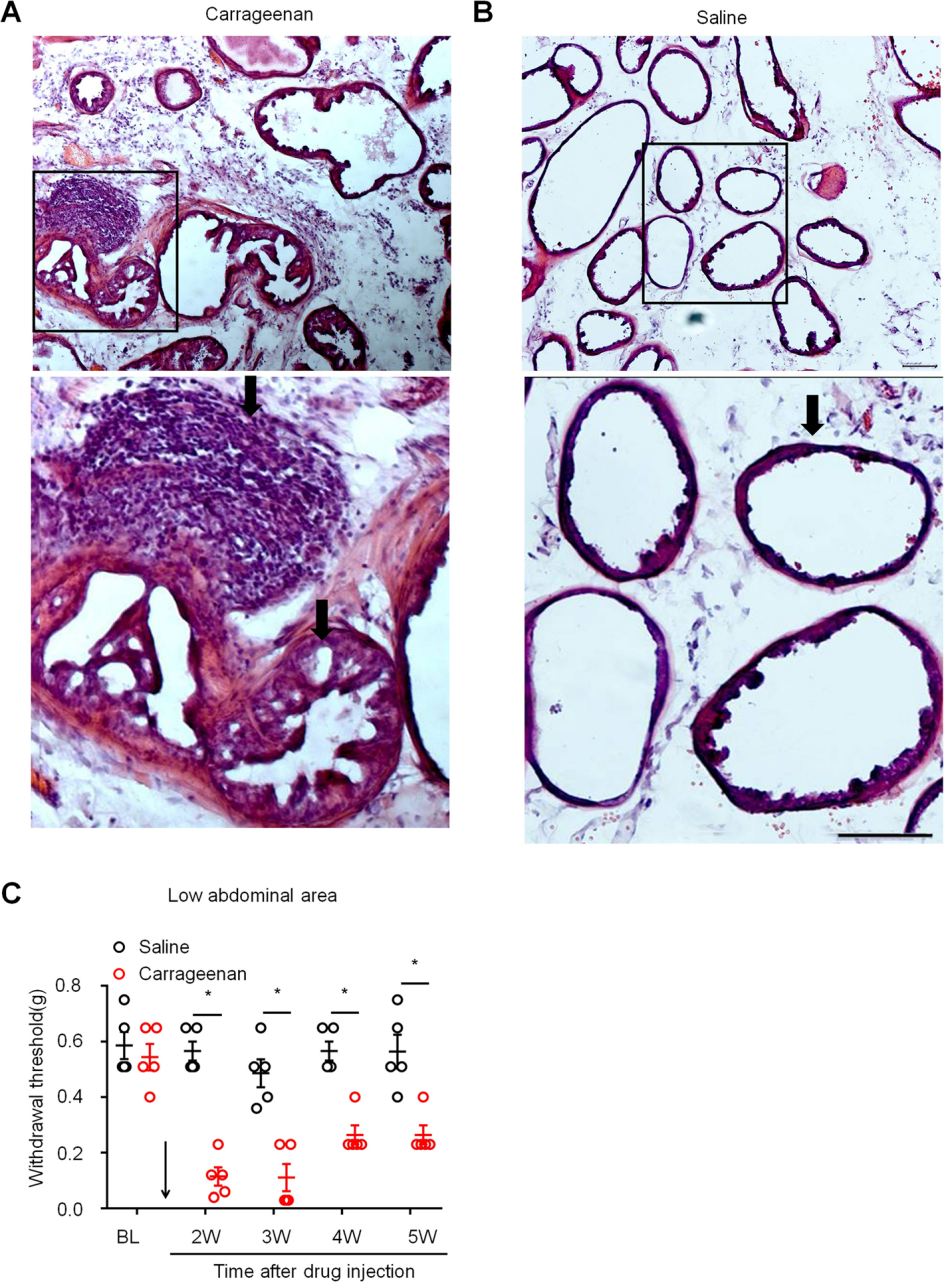
Carrageenan injection into the prostate induced chronic prostatitis and mechanical hyperalgesia. **a** Images of HE staining showing lots of inflammatory cells infiltrated in the prostate stroma 5 weeks after carrageenan injection. **b** Images of HE staining showing no inflammatory cell infiltration was observed in the prostatic stroma in the saline-treated group. Top: low-magnification image, bottom: enlarged images from the box. Scales, 100 μm. **c** Carrageenan-induced mechanical allodynia in the lower abdominal area. BL, baseline. *P < 0.05, vs. BL (baseline). Two-way ANOVA, followed by post hoc Bonferroni test. *n* = 5 mice in each group. All data are expressed as mean ± S.E.M.

Pathological examination by hematoxylin-eosin (HE) staining showed that a large number of inflammatory cells infiltrated the prostate stroma 5 weeks after carrageenan injection into the prostate, and some prostate mucosal cells showed degeneration, necrosis, and shedding (Fig. 2a). In the control group, no inflammatory cell infiltration was observed in the prostatic stroma, and the mucosal epithelial cells retained their normal arrangement (Fig. 2b). Quantitation of the degree of inflammation in prostate is shown in Additional file 1: Table S2. These results indicated that a mouse model of CP/CPPS was successfully established after carrageenan injection into the prostate.

Next, we measured prostatitis-induced chronic pain by measuring the mechanical sensitivity of the lower abdominal area in carrageenan- and saline-treated mice. Since direct measurement of pain in the prostate gland is not possible in freely moving animals, the escape behavior of the animals in response to mechanical stimulus related to “visceral pain” has been verified by previous report [19]. Comparison of the CP/CPPS model mice and control mice injected with saline revealed that mechanical allodynia was fully developed at 2 weeks and persisted for at least 5 weeks after carrageenan injection into the prostate of the mice (Fig. 2c, *P* < 0.05 versus BL, two-way ANOVA, followed by post hoc Bonferroni test), indicating that persistent prostate pain was induced after carrageenan injection.

### Activation of spinal astrocytes but not microglia in the late phase of prostatitis

**Fig. 3:**
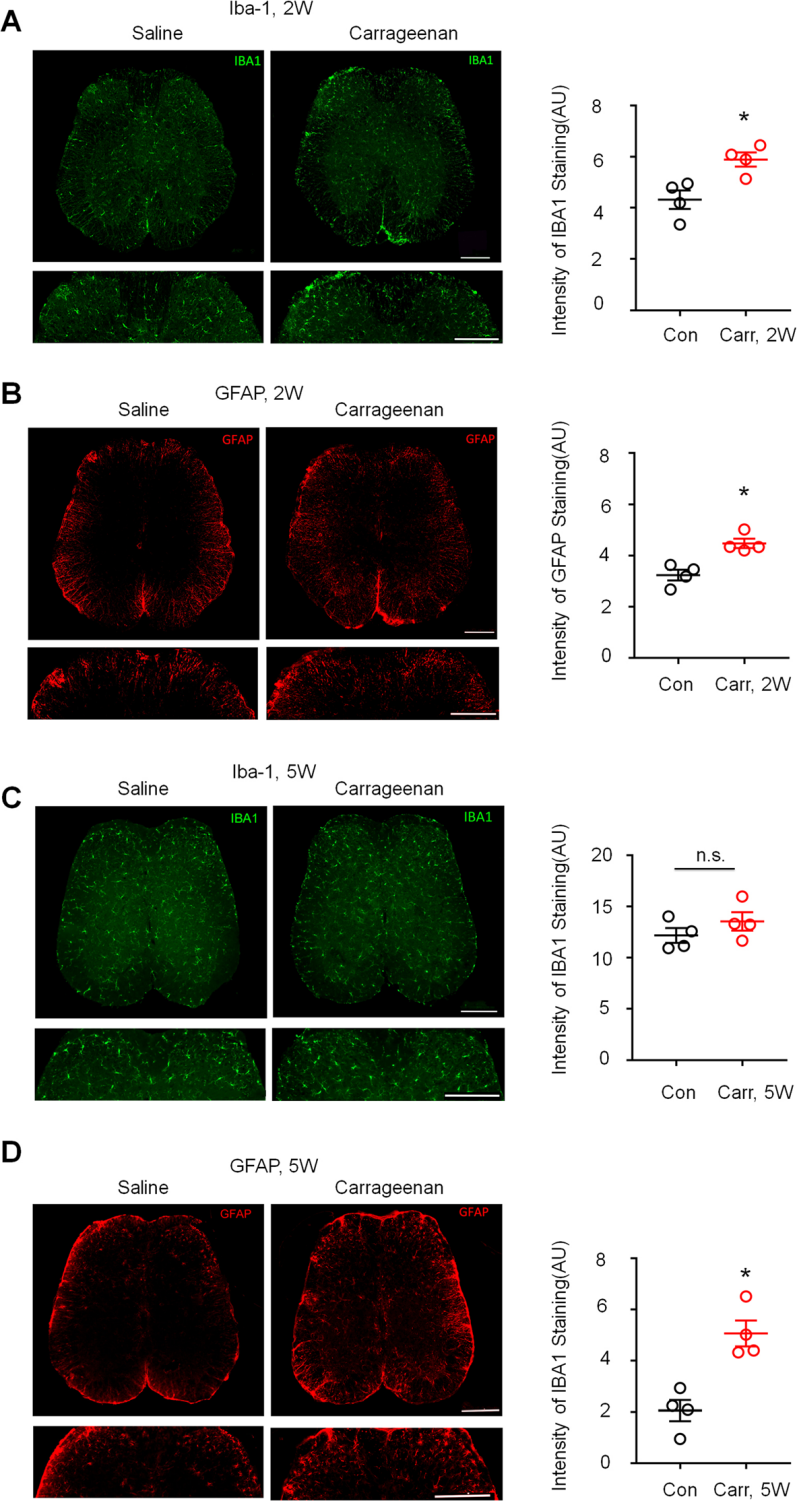
Activation of spinal astrocytes but not microglia in the late-phase of prostatitis. **a**, **b** Left: images of Iba-1 (**a**) and GFAP (**b**) immunostaining showing carrageenan-induced Iba-1 expression in the spinal cord dorsal horn 2 weeks after injection compared with the saline-treated group. Scales, 200 μm in top and bottom panels. Right: quantification of the intensity of Iba-1 and GFAP immunostaining in the carrageenan-treated group and the saline-treated group. *P < 0.05, vs. saline, Student’s *t* test. *n* = 4 mice/group. **c**, **d** Left: images of Iba-1 (**a**) and GFAP (**b**) immunostaining showing carrageenan-induced Iba-1 expression in the spinal cord dorsal horn 5 weeks after injection compared with the saline-treated group. Scales, 200 μm in top and bottom panels. Right: quantification of the intensity of Iba-1 and GFAP immunostaining in the carrageenan-treated group and the saline-treated group. n.s., not significant. *P < 0.05, vs. saline, Student’s *t* test. *n* = 4 mice/group. All data are expressed as mean ± S.E.M.

To determine whether CP/CPPS activates spinal microglia and astrocytes, we examined the expression of the microglia marker Iba-1 and astrocyte marker glial fibrillary acidic protein (GFAP) in L6~S1 lumber cord sections after carrageenan injection into the prostate. Because mechanical allodynia caused by the carrageenan-injected CP/CPPS model was obvious at approximately 2 weeks and lasted for more than 5 weeks, we examined spinal microglia and astrocytes 2 weeks and 5 weeks after carrageenan injection, corresponding to the early and late stages of prostatodynia, respectively. Immunostaining showed that the expression of Iba-1 and GFAP in the L6–S1 spinal cord dorsal horn was higher in the carrageenan-injected group than in the saline-treated group at 2 weeks (Fig. 3a, b, *P* < 0.05 versus control, Student’s *t* test). Furthermore, at 5 weeks after carrageenan injection, the expression of GFAP in the L5–S1 spinal cord dorsal horn was still higher than that in the saline-treated group (Fig. 3d, *P* < 0.05 versus control, Student’s *t* test), but there was no difference in Iba-1 expression between the two groups at the later time point (Fig. 3c).

**Fig. 4:**
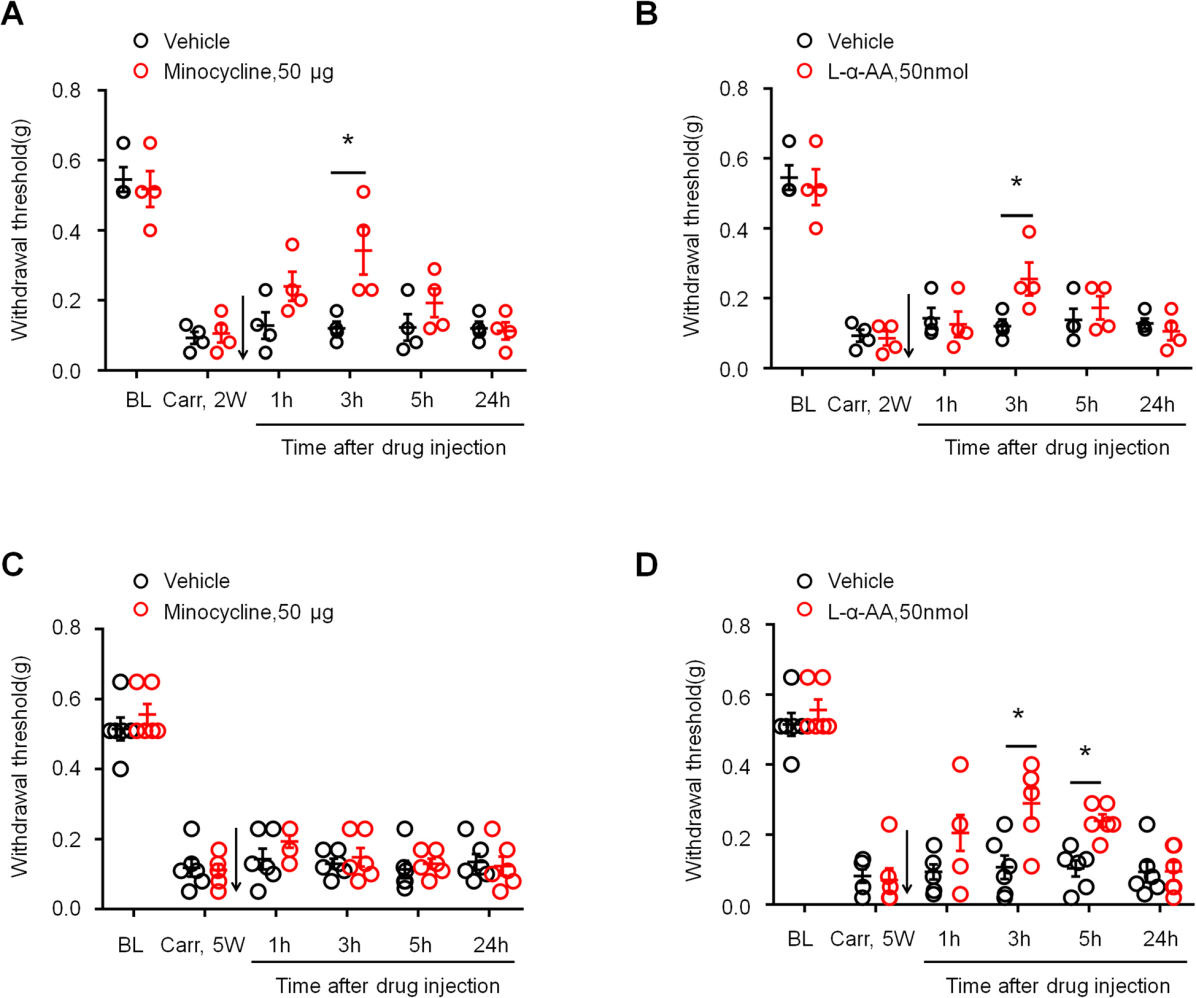
Effects of intrathecal injection of microglial inhibitor and astroglial toxin on carrageenan-induced early- and late-phase mechanical allodynia in prostatitis mice. **a**, **b** Prostatitis-induced mechanical hypersensitivity in the lower abdominal area at 2 weeks was partially reversed by spinal injection of minocycline (50 μg) and L-AA (50 nmol). **c**, **d** Prostatitis-induced mechanical hypersensitivity in the lower abdominal area at 5 weeks was not reversed by spinal injection of minocycline (50 μg), but partially reversed and L-AA (50 nmol). BL, baseline. *P < 0.05, vs. Carr 2w or Carr 5w, two-way ANOVA, followed by post hoc Bonferroni test. *n* = 6 mice/group. All data are expressed as mean ± S.E.M. Arrows indicate drug injection

To further investigate the roles of spinal astrocytes and microglia in late-phase prostate pain, mice with CP/CPPS were given the astroglial toxin L-2-Aminoadipate acid (L-AA; 50 nmol, i.t.) and the microglial inhibitor minocycline (50 μg, i.t.) 2 and 5 weeks after carrageenan injection. At 2 weeks, mechanical hypersensitivity was completely reversed within 1 h after intrathecal injection of minocycline and L-AA, and the reversal effect lasted for more than 5 h and disappeared after 24 h (Fig. 4a, b, *P* < 0.05 versus vehicle, two-way ANOVA, followed by post hoc Bonferroni test), which suggested that chronic prostatitis-induced microglia and astrocyte activation contributes to pain hypersensitivity. However, intrathecal injection of L-AA but not minocycline reduced mechanical allodynia at 5 weeks after CP/CPPS, indicating that spinal astroglial but not microglial signaling is the major reason for persistent mechanical allodynia in the late stage of CP/CPPS (Fig. 4c, d, in Fig. 4d, *P* <  0.05 versus vehicle, two-way ANOVA, followed by post hoc Bonferroni test).

### Carrageenan induces ERK activation in the spinal cord dorsal horn associated with prostatitis pain

**Fig. 5:**
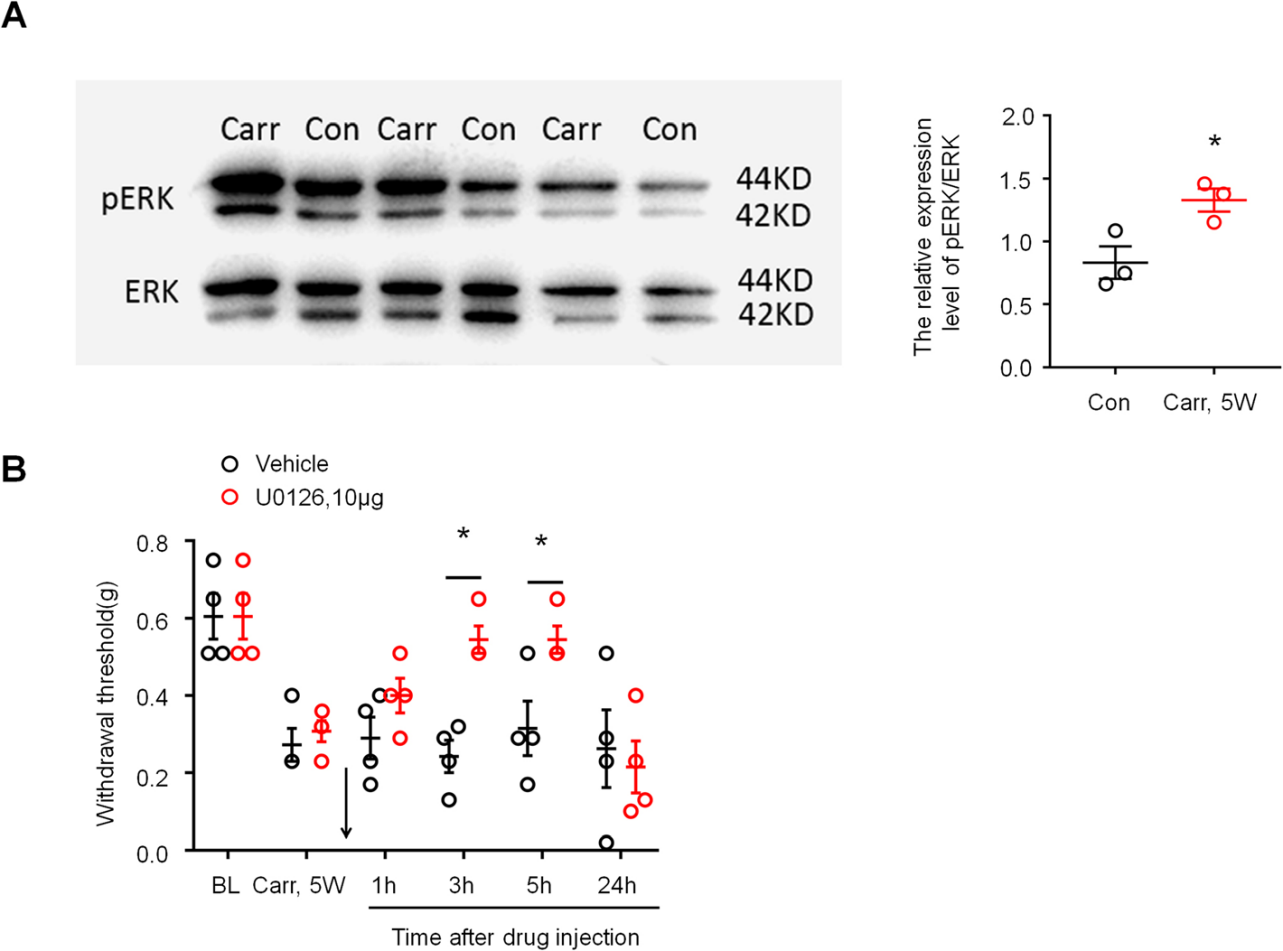
Prostatitis induced ERK activation in spinal cord dorsal horn and contributed to mechanical allodynia. **a** Western blotting shows that prostatitis induced pERK expression in SDH 4 weeks after carrageenan injection into the prostate. Right, quantification of pERK levels in SDH in the carrageenan-treated group and the saline-treated group. *P < 0.05, vs. control (con, saline-treated group), Student’s *t* test. *n* = 4 mice/group. **b** Inhibition of prostatitis-induced mechanical allodynia in the lower abdominal area by intrathecal injection of U0126 (10 μg) at 5 weeks. BL, baseline. *P < 0.05, vs. Carr 5w, two-way ANOVA, followed by post hoc Bonferroni test. *n* = 6 mice/group. All data are expressed as mean ± S.E.M. Arrows indicate drug injection

Accumulating evidence suggests that ERK phosphorylation in activated astrocytes plays an essential role in the maintenance of inflammatory and neuropathic pain models in mice. We therefore reasoned that phosphorylated ERK (pERK) may also be associated with the maintenance of prostatitis pain. We first compared the activation of ERK in the spinal cord dorsal horn of saline-treated and carrageenan-treated mice by Western blot. As shown in Fig. 5a, carrageenan elicited marked pERK expression in the spinal cord dorsal horn at 5 weeks after injection (*P* < 0.05 versus control, Student’s *t* test). Then, we examined whether carrageenan induced persistent prostate pain behavior via ERK by intrathecal injection of the ERK kinase inhibitor U0126. As expected, intrathecal U0126 (10 μg) completely reversed mechanical allodynia in the lower abdominal area 3 h after injection, and the reversal effect lasted for more than 5 h and disappeared after 24 h (Fig. 5b, *P* < 0.05 versus vehicle, two-way ANOVA, followed by post hoc Bonferroni test). These results suggested that ERK is critically involved in carrageenan-induced persistent prostate pain.

### Cx43 plays an important role in maintaining the chronic pain caused by prostatitis

**Fig. 6:**
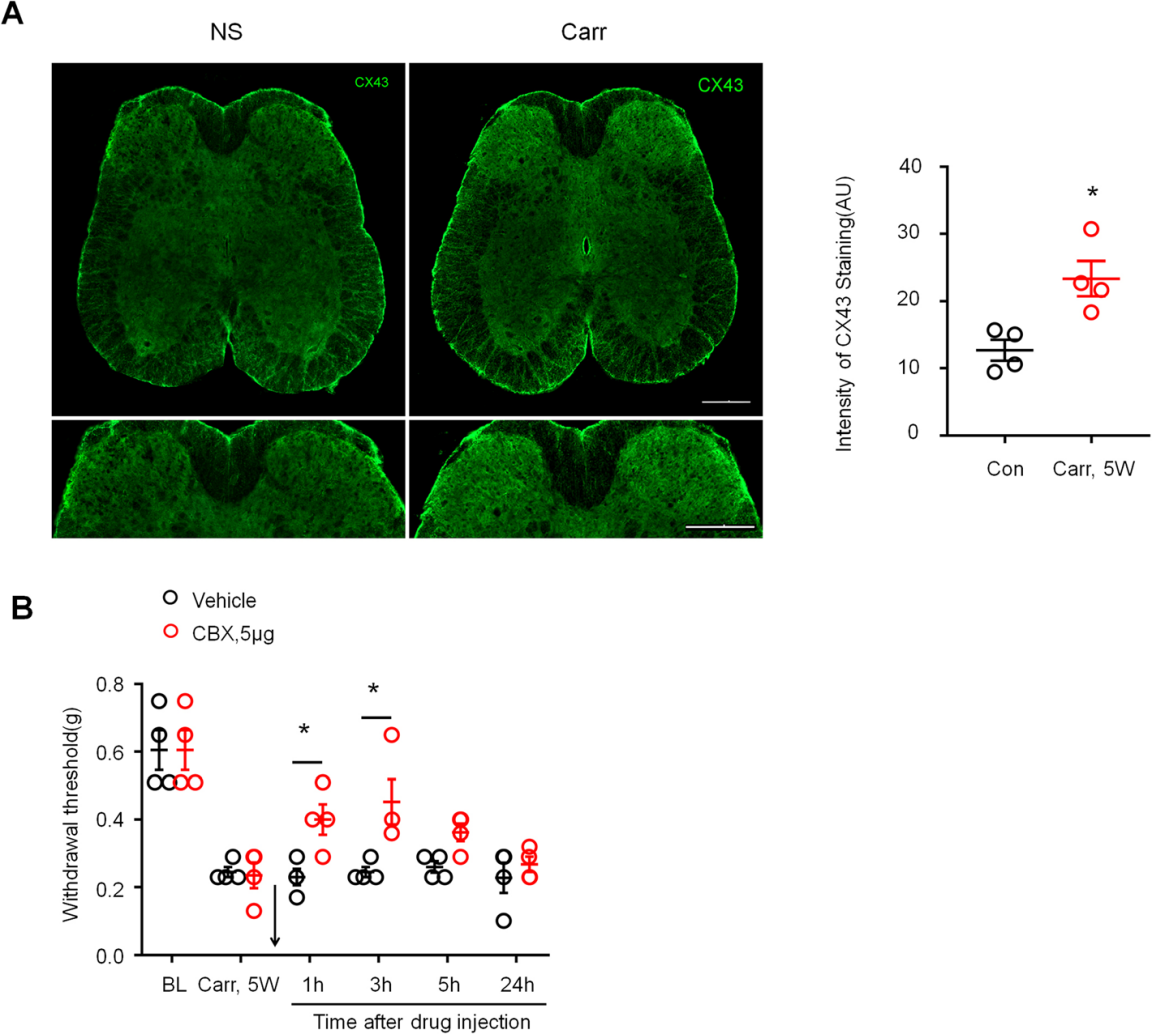
Prostatitis induced Connexin43 expression in spinal cord dorsal horn and contributed to mechanical allodynia. **a** Prostatitis-induced Cx43 expression in SDH 5 weeks after carrageenan injection into the prostate. Scales, 200 μm in top and bottom panels. Right, quantification of the intensity of Cx43 immunostaining in the carrageenan-treated group and the saline-treated group. *P < 0.05, vs. control (con, saline-treated group), Student’s *t* test. *n* = 4 mice/group. **b** Inhibition of prostatitis-induced mechanical allodynia in the lower abdominal area by intrathecal injection of CBX (5 μg) at 5 weeks. BL, baseline. *P < 0.05, vs. Carr 5w, two-way ANOVA, followed by post hoc Bonferroni test. *n* = 6 mice/group. All data are expressed as mean ± S.E.M. Arrows indicate drug injection

Recent studies have shown an emerging role of astrocyte Cx43 in chronic pain [25]. Therefore, we speculated that Cx43 plays a role in maintaining the chronic pain caused by prostatitis. First, we examined the expression of CX43 in L6~S1 lumber cord sections 5 weeks after carrageenan injection. Immunostaining showed that carrageenan induced a significant increase in Cx43 expression in the spinal dorsal horn compared to saline injection (Fig. 6a, *P* < 0.05 versus control, Student’s *t* test). Next, we tested whether CBX, a nonselective Cx43 inhibitor, could reverse the mechanical allodynia in the lower abdominal area caused by prostatitis. As shown in Fig. 6b, intrathecal injection of CBX (5 μg) rapidly and completely reversed mechanical allodynia in the lower abdominal area, and the reversal effect lasted for more than 3 h and disappeared after 24 h (*P* < 0.05 versus vehicle, two-way ANOVA, followed by post hoc Bonferroni test).

### CXCL1 signaling contributes to the chronic pain caused by prostatitis

**Fig. 7:**
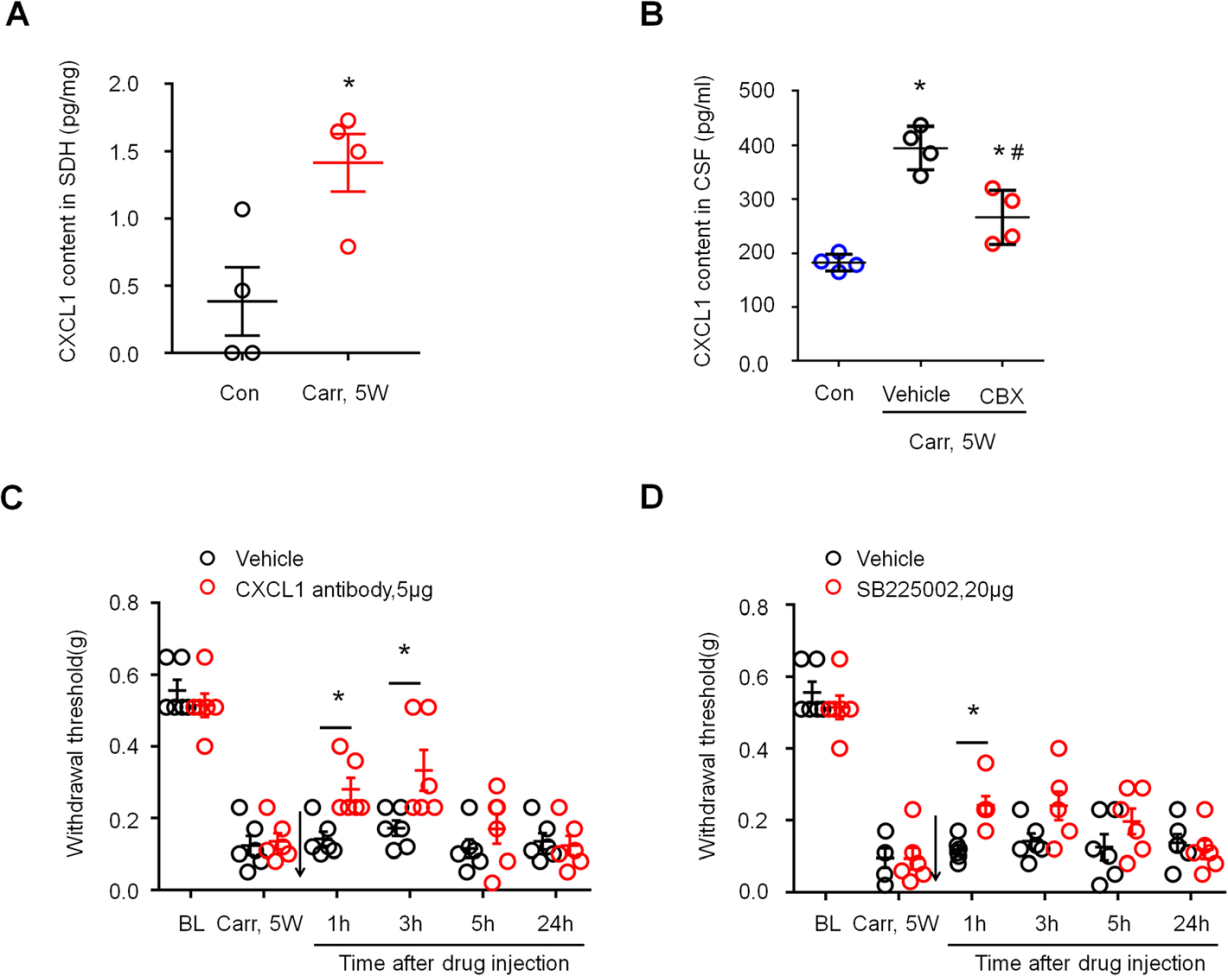
Prostatitis induced CXCL1 expression in spinal cord dorsal horn and contributed to mechanical allodynia. **a** ELISA analysis showing CXCL1 expression was increased in spinal cord dorsal horn 5 weeks after carrageenan injection. *P < 0.05, vs. control (con, saline-treated group), Student’s *t* test. *n* = 4 mice per group. **b** ELISA analysis shows decreased CXCL1 release in the CSF in the carrageenan-injected group at 3 h after the intrathecal injection of CBX (5 μg). *P < 0.05, vs. control (con, saline-treated group); ^#^*P* < 0.05, vs. the vehicle group. One-way ANOVA, followed by post hoc Bonferroni test. *n* = 4 mice/group. **c** Prostatitis-induced mechanical allodynia in the lower abdominal area were partially reversed by intrathecal injection of the CXCL1 neutralizing antibody (5 μg) at 5 weeks after carrageenan injection. **d** Prostatitis-induced mechanical allodynia in the lower abdominal area was partially reversed by intrathecal injection of the CXCR2 antagonist SB225002 (20 μg) at 5 weeks after carrageenan injection. BL, baseline. *P < 0.05, vs. Carr 5w, two-way ANOVA, followed by post-hoc Bonferroni test. *n* = 6 mice/group. All data are expressed as mean ± S.E.M. Arrows indicate drug injection

Previous reports have shown that Cx43 controls the astrocytic release of CXCL1 [16, 25], and CXCL1 contributes to chronic pain by activating CXCR2 receptors [26]. To demonstrate whether a similar molecular mechanism exists in prostatitis pain, we first examined the expression of CXCL1 in the spinal cord dorsal horn 5 weeks after carrageenan injection. The ELISA results showed significantly higher CXCL1 expression in the carrageenan-injected group than in the saline-injected group (Fig. 7a, *P* < 0.05 versus control, Student’s *t* test). To investigate the effect of CBX on the secretion of CXCL1 in a mouse model of carrageenan-induced prostatitis, we compared CXCL1 levels in cerebrospinal fluid (CSF) in the saline-injected group, the PBS-treated carrageenan injection group, and the CBX-treated carrageenan injection group. ELISA analysis showed an increase in CXCL1 levels in the CSF of the carrageenan injection group compared to the saline injection group. CXCL1 levels in the CSF of the carrageenan injection group were significantly reduced 3 h after the intrathecal injection of CBX (5 μg) (Fig. 7b, *P < 0.05, compared with control (saline-treated group); ^#^*P* < 0.05, compared with the vehicle group; one-way ANOVA, followed by post hoc Bonferroni test). We next tested whether blocking CXCL1 signaling could attenuate the mechanical hypersensitivity caused by prostatitis. Intrathecal injection of a CXCL1 neutralizing antibody (5 μg) significantly reduced mechanical allodynia in the lower abdominal area for > 3 h (Fig. 7c, *P* < 0.05 versus vehicle, two-way ANOVA, followed by post hoc Bonferroni test). CXCR2 is a major receptor of CXCL1, and intrathecal injection of the CXCR2 antagonist SB225002 (20 μg) also significantly reduced mechanical allodynia in the lower abdominal area for > 1 h (Fig. 7d, *P* < 0.05 versus vehicle, two-way ANOVA, followed by post hoc Bonferroni test). These results suggest that CXCL1 signaling contributes to the mechanical allodynia caused by prostatitis.

## Discussion

In order to study prostatitis in the future using transgenic mice, a mouse model of carrageenan-induced chronic prostatitis was established in this study. To the best of our knowledge, this is the first report of carrageenan-induced prostatitis in mice. According to the literature, in the rat model, injection of carrageenan with a concentration ranging from 1 to 3% and a volume from 20 to 100 μl can successfully induce prostatitis (1%, 20 μl: [27, 28]; 1%, 100 μl: [29, 30]; 2%, 50 μl: [31]; 3%, 25 μl: [19]; 3%, 50 μl: [32]; 3%, 100 μl [33]). We used histological examination to compare mouse models of different concentrations (1%, 2%, and 3%) and volumes (20 or 50 μl) of carrageenan-induced prostatitis (data not shown). We found that injection of carrageenan (1%, 20 μl) into both right and left ventral lobes of the prostate in mouse can successfully establish a model of chronic prostatitis.

The mechanism of chronic pain caused by prostatitis is still unclear. In this study, we found that a novel mechanism of activated spinal astrocytes plays a crucial role in maintaining chronic prostatitis-induced persistent pain. The mechanism was dissected by a variety of experimental methods. First, activation of spinal astrocytes but not microglia was found in the late phase of CP/CPPS. Second, intrathecal injection of the astroglial toxin L-AA and pERK inhibitor effectively alleviated the symptoms of mechanical allodynia. Third, CP/CPPS induced a profound and persistent upregulation of Cx43 hemichannels in spinal cord astrocytes, and spinal injection of CBX effectively reduced pain symptoms and the content of CXCL1 in CSF. Fourth, increased expression of CXCL1 was found in the spinal dorsal horn in the late stage of CP/CPPS. Finally, intrathecal injection of a CXCL1 neutralizing antibody or CXCR2 (a major receptor of CXCL1) antagonist SB225002 significantly reduced mechanical allodynia in the late phase of CP/CPPS.

### Visceral-related pain and spinal cord sensitization

Several studies have shown that neural mechanisms of spinal cord sensitization are an important factor associated with the pathogenesis of visceral-related pain. Visceral inflammation increases neurotransmitter levels and neuronal activity in the afferent pathway. For example, microglia activation and increased tumor necrosis factor (TNF)-α production were found in the hippocampus of an experimental bowel inflammation model, and these changes resulted in increased central nervous system (CNS) excitability [34]. Furthermore, intrathecal injection of minocycline [35] or the TNF-α antibody [36] reduced pain behaviors in animal models of inflammatory bowel disease. Endometriosis-induced chronic pelvic pain involves neuronal processes leading to central sensitization [37]. The expression of phosphorylated p38-mitogen-activated protein kinase (MAPK) in spinal microglia [38] or in the rostral ventromedial medulla [39] was increased following endometriosis in rats, and this increase was essential for central sensitization. In a rat model of CP/CPPS, afferent electrical activity of the pelvic nerve was significantly enhanced compared with that in the control group [40]. Prostatitis causes inflammation in the spinal cord and activation of spinal microglia [11]. Previous reports have found that the levels of some molecules associated with pain conditions, including CCL3, IL-1B, TNF-α, brain-derived neurotrophic factor (BDNF), and substance P, are increased in the spinal cord of prostatitis animal models [11, 41]. Moreover, a recent study has shown higher levels of IL-1β and IL-6 in the thalamus and cortex in CP/CPPS rats compared to Sham [32]. The present study showed that carrageenan injection induced persistent prostate pain in 2 weeks that lasted for more than 6 weeks. Activation of spinal microglia and astrocytes was increased in the L6 to S2 spinal cord segments and contributed to mechanical allodynia. Furthermore, the synthesis and secretion of the chemokine CXCL1, a mediator of neuroinflammation, in the spinal dorsal horn is increased in the prostatitis model. Our results support the hypothesis that CP/CPPS can cause spinal cord sensitization.

### Spinal astrocytes, ERK, Cx43, and late-phase prostatitis pain

Accumulating evidence has shown that astrocytes play an important role in spinal cord sensitization and contribute to a variety of types of chronic pain. Various signaling molecules are upregulated in activated astrocytes, including cell-surface receptors, transports, protein kinases, and secreted signaling molecules, which coordinate or interact with each other in response to pain. One of the important findings of this study is that activation of spinal astrocytes but not microglia in the late phase (5 weeks) of prostatitis and intrathecal injection of L-AA but not minocycline reduced mechanical allodynia at 5 weeks after CP/CPPS. Our results indicate that activation of astrocytes is a major reason for the persistent pain caused by prostatitis.

Activation of ERK (extracellular signal-regulated kinase 1 and 2) is a critical step of astrocyte activation in the spinal cord after noxious stimulation and contributes to the induction and maintenance of persistent pain [14, 15, 42]. Prevention of ERK activation with MEK inhibitors can significantly reduce inflammatory pain by inhibiting both peripheral and central sensitization [42–45]. The present study demonstrated that carrageenan injection can cause a significant increase in pERK expression in the dorsal horn of the spinal cord and intrathecal injection of the ERK kinase inhibitor U0126 completely reversed carrageenan-induced mechanical allodynia. These results suggested that ERK is critically involved in carrageenan-induced persistent prostate pain.

Another interesting finding is that the upregulation of Cx43 contributes to prostatitis-induced persistent pain by enhancing the chemokine CXCL1 production and release. Cx43 is the predominant connexin expressed by astrocytes and not only acts as a non-ligated hemichannel to release small molecules and ions but also increases the expression and secretion of cytokines and chemokines in some types of cells [25], including bone marrow stromal cells [46], fibroblast-like synoviocytes [47], and astrocytes [16, 48]. For example, activated astrocytes can release multiple inflammatory mediators and neuromodulators, such as cytokines (interleukin (IL)-1b) and chemokines (C-C motif ligand 2 (CCL2) and CXCL1), via the upregulation of the hemichannel and gap junction protein Cx43 to enhance and extend persistent pain states [16]. The present study showed that prostatitis induced a profound and persistent upregulation of Cx43 in spinal astrocytes, and intrathecal injection of CBX effectively reduced prostatitis pain and the content of CXCL1 in CSF. CXCL1, as a proinflammatory chemokine, has been shown to maintain neuropathic pain in various models of chronic pain. Here, the critical role of CXCL1 signaling in mechanical allodynia caused by prostatitis was demonstrated by several lines of evidence, including increased expression of CXCL1 in the spinal cord dorsal horn and attenuation of mechanical hypersensitivity by intrathecal injection of the CXCL1 neutralizing antibody or CXCR2 antagonist. Our results demonstrate that activated astrocytes contribute to the later phase of carrageenan-induced prostatitis pain via Cx43-regulated CXCL1 production and secretion.

The relationship between ERK and Cx43 has been reported in some literature. For example, Cx43 peptide or siRNA treatment significantly inhibited FGF10-induced increase in ERK1/2 phosphorylation in salivary gland epithelial cell line HSY [49]. The ERK inhibitor PD98059 prevented the analogue of combretastatin A-4 (HYS-32)-induced increase in Cx43 expression in rat astrocytes [50]. Interestingly, Cx43 inhibitors reduced ERK1/2 activation and ERK inhibitor PD98059 also attenuated Cx43 expression in cultured human umbilical vein endothelial cells [51]. Furthermore, in a rat model of traumatic brain injury, enhanced expression of phosphorylated Cx43 and ERK was found to be located in the same astrocyte [52]. These results suggest that there is a positive correlation between Cx43 and ERK.

## Conclusions

In this study, we have demonstrated that activated spinal astrocytes play a crucial role in maintaining chronic prostatitis-induced persistent pain via Cx43-regulated CXCL1 production and secretion. Prostatitis also causes an increase in ERK phosphorylation in the spinal cord dorsal horn, and intrathecal injection of ERK inhibitor can significantly reduce prostatitis-induced mechanical allodynia.

### Clinical relevance

Prostatitis pain is the main symptom of CP/CPPS. At present, clinical treatments of prostatitis pain mainly use antibiotics, anti-inflammatory drugs, alpha blockers, botulinum toxin, acupuncture, and shock therapy [7]. Unfortunately, these therapies usually target inflammation of the prostate, and lack of treatment of the increased CNS sensitivity associated with prostatitis has limited the effectiveness of pain relief. Patients need more effective treatment methods. Given the important roles of astrocytes in the maintenance of prostatitis pain, astrocyte-targeting drugs may help alleviate persistent pain. Studies that further investigate the pathological changes of astrocytes associated with prostatitis are desirable. Such information would enhance our knowledge of prostatitis pain mechanisms and help identify more effective treatments with fewer side effects.

## Supplementary information

**Additional file 1: Figure S1**. Surgical picture of carrageenan or saline injection. **Figure S2**. Scheme presenting an overview over the measured region. **Figure S3**. Double immunostaining of IBA1 and F4/80 in spinal cord of mice. **Figure S4**. Full size western blots of Fig. 5a. **Table S1**. Information of mice used in different experiments. **Table S2**. The proposed classification of prostatic inflammatory infiltrates.

## Data Availability

Please contact the author for data requests.

## Abbreviations

BDNF: Brain-derived neurotrophic factor
CBX: Carbenoxolone
CNS: Central nervous system
CP/CPPS: Chronic prostatitis
CSF: Cerebrospinal fluid
Cx43: Connexin-43
CXCL1: C-X-C motif ligand 1
ERK: Extracellular signal-regulated kinase
GFAP: Glial fibrillary acidic protein
HE: Hematoxylin-eosin
L-AA: L-2-Aminoadipate acid
SDH: Spinal cord dorsal horn
TNF: Tumor necrosis factor
