# Supplementary material for: Activated spinal astrocytes contribute to the later phase of carrageenan-induced prostatitis pain
Source: J Neuroinflammation. 2019 Oct 25;16:189. doi: 10.1186/s12974-019-1584-3 (PMC6814979; doi:10.1186/s12974-019-1584-3)
Supplement: Supplementary file 1 — Additional file 1: Figure S1. Surgical picture of carrageenan or saline injection. Figure S2. Scheme presenting an overview over the measured region. Figure S3. Double immunostaining of IBA1 and F4/80 in spinal cord of mice. Figure S4. Full size western blots of Fig. 5a. Table S1. Information of mice used in different experiments. Table S2. The proposed classification of prostatic inflammatory infiltrates. [file 12974_2019_1584_MOESM1_ESM.docx]

**Activated spinal astrocytes contribute to the later phase of carrageenan-induced** **prostatitis pain**

Guo-Chuang Deng, Ming-Lu, Ya-Yu Zhao, Ying Yuan, Gang Chen

**Figure S1.** Surgical picture of carrageenan or saline injection.

**
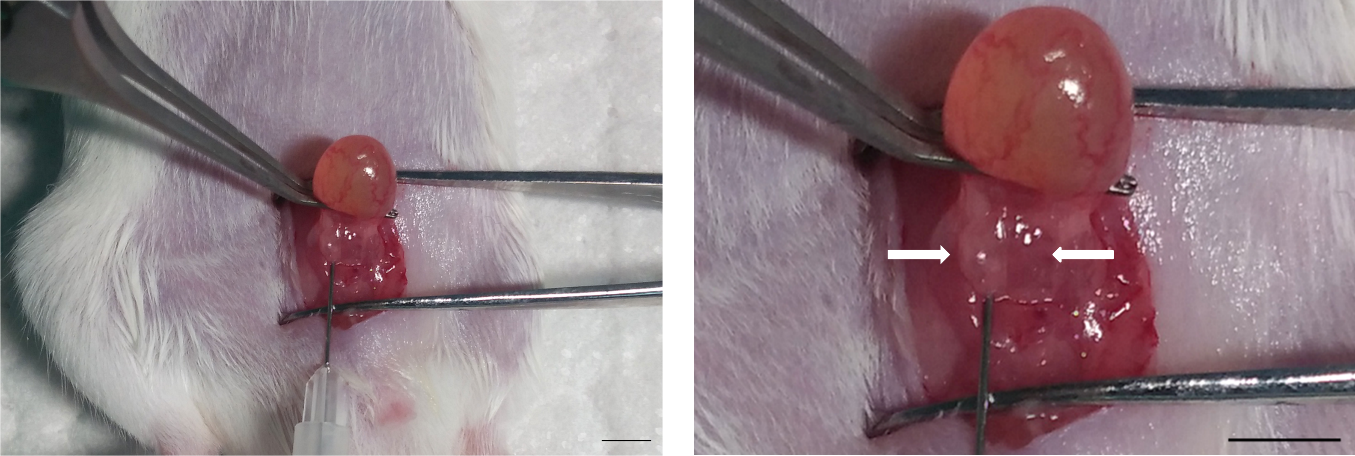
**

Figure legend: Bilateral injection of 1% carrageenan solution (20 μl) or saline (20 μl) into the CD-1 mouse prostate lobes. Scale bars: 5 mm.

**Figure S2.** Scheme presenting an overview over the measured region.

**
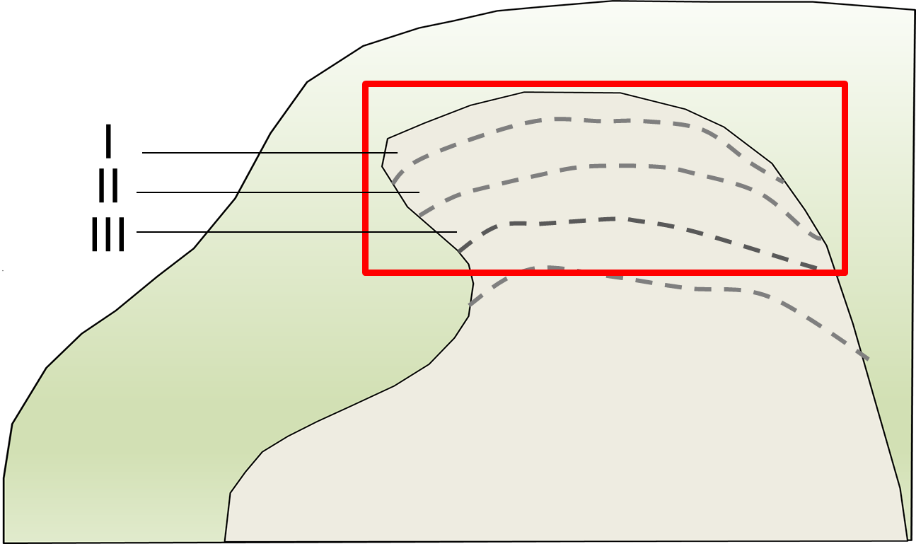
**

Figure legend: The intensity of fluorescence in spinal cord superficial dorsal horn (laminae I–III) was measured by a blinded observer using NIH Image J software from a rectangular region (red box).

**Figure S3. Double immunostaining of IBA1 and F4/80 in spinal cord of mice.**

**
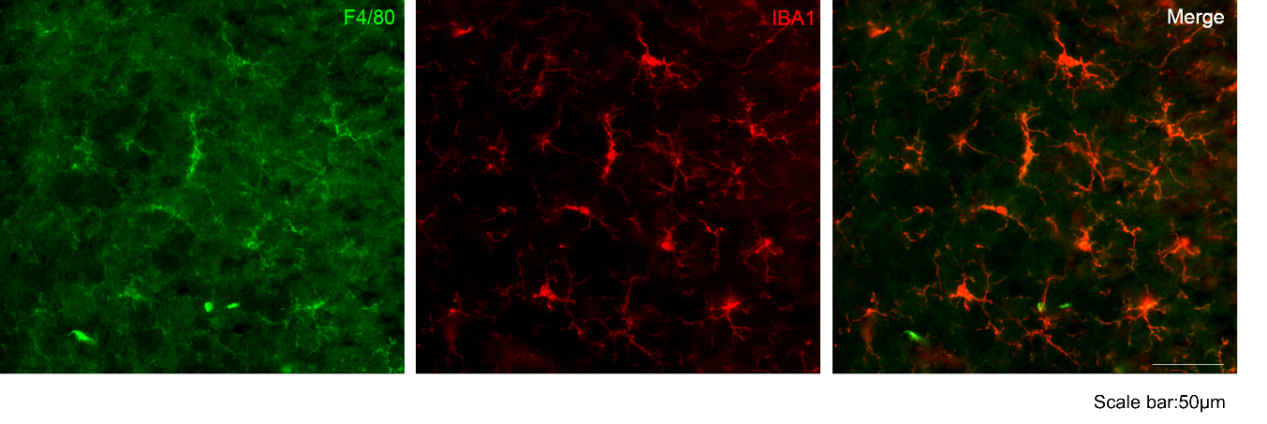
**

Figure legend: Images of F4/80 (green) and IBA1 (red) immunostaining showing carrageenan-induced microglial activation in the spinal cord dorsal horn 2 weeks after injection. Scales, 50 µm.

**Figure S4.** Full size western blots of Figure 5a.


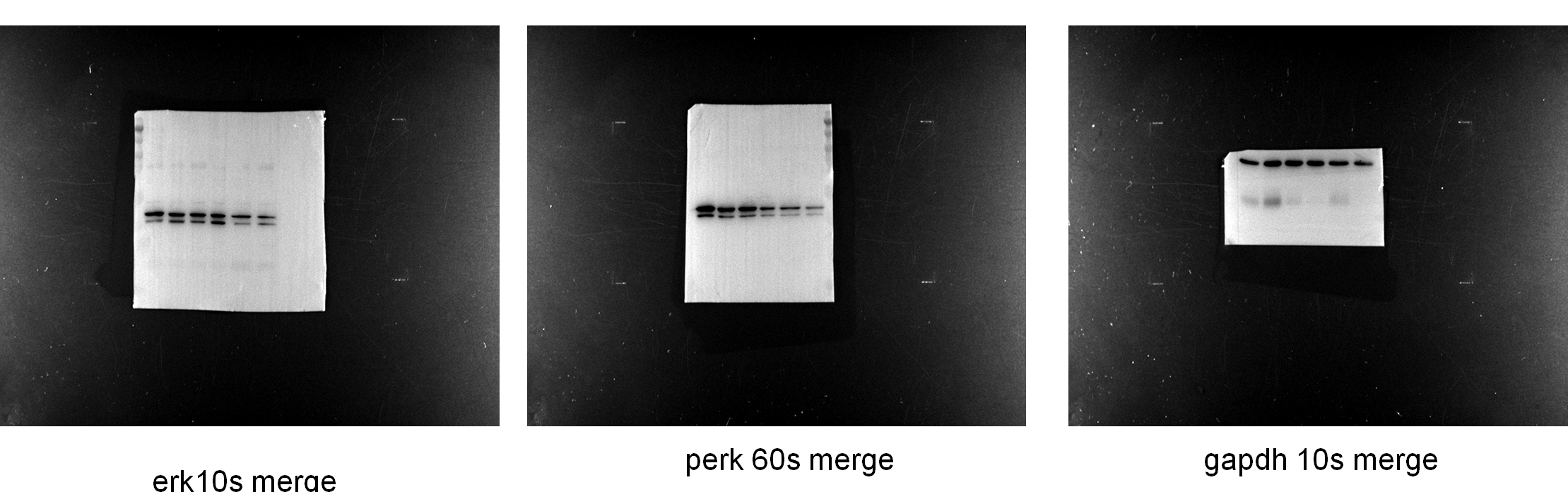


**Table S1: Information of mice used in different experiments.**

| Experiment | Number of groups | Sample size | Sex | Ages | Number of mice |
| --- | --- | --- | --- | --- | --- |
| HE staining | 2 | n=5 mice | male | 8 weeks | 10 |
| Behavioral test | 12 | n=4-6 mice | male | 8 weeks | 58 |
| Immunohistochemistry | 4 | n=4 mice | male | 8 weeks | 16 |
| Wetern blot | 2 | n=4 mice | male | 8 weeks | 8 |
| Elisa | 5 | n=4 mice | male | 8 weeks | 20 |
| Prostate indexes | 2 | n=4 mice | male | 8 weeks | 8 |
|  | Total number of mice | | | | 120 |

A total of 120 male mice (8 weeks) were used in this study, 93 mice underwent carragenenan injection and 25 mice underwent saline injection.

**Table S2: The proposed classification of prostatic inflammatory infiltrates.**

| Inflammation grading situation for prostatitis mice | | | | | |
| --- | --- | --- | --- | --- | --- |
| Carrageenan group | | | | | |
| Grade | 1/mild(%) | 2/moderate(%) | 3/severe(%) | prostatitis mice | Total number of mice |
| Glandular | 0(0) | 5(50) | 5(50) | 10 | 10 |
| Periglandular | 0(0) | 4(40) | 6(60) | 10 | 10 |
| Stromal | 0(0) | 5(50) | 5(50) | 10 | 10 |
| Saline group | | | | | |
| Grade | 1/mild(%) | 2/moderate(%) | 3/severe(%) | prostatitis mice | Total number of mice |
| Glandular | 0(0) | 0(0) | 0(0) | 0 | 10 |
| Periglandular | 0(0) | 0(0) | 0(0) | 0 | 10 |
| Stromal | 0(0) | 0(0) | 0(0) | 0 | 10 |

Note: The standard for grade morphological description (typical inflammatory cell density, cells/mm^2^) comes from the literature (Nickel et al., BJU Int 2001, 87:797-805).

1/mild：individual inflammatory cells, most of which are separated by distinct intervening spaces (<100)

2/moderate：confluent sheets of inflammatory cells with no tissue destruction or lymphoid nodule/follicle formation (100±500)

3/severe：confluent sheets of inflammatory cells with tissue destruction or nodule/follicle formation (>500)
